# Early Childhood Caries and Its Association With Behavior in Preschool Children

**DOI:** 10.7759/cureus.58648

**Published:** 2024-04-20

**Authors:** Riddhi R Asundaria, Shruthi B Patil

**Affiliations:** 1 Dentistry, ToothFables Clinic, Mumbai, IND; 2 Paediatric and Preventive Dentistry, Sri Dharmasthala Manjunatheshwara (SDM) College of Dental Science and Hospital, Dharwad, IND

**Keywords:** child behavior checklist (cbcl), behavior, dental caries, preschool children, early childhood caries

## Abstract

Background and aim

Early childhood caries (ECC) is a profoundly impactful multifactorial condition that not only influences a child’s overall well-being but also diminishes their quality of life. Given the limited availability of literature on the relationship between children’s behavior and ECC, the present study utilized a standardized assessment tool to assess the association between ECC and behavioral changes in preschool children.

Methodology

Our study cohort consisted of 120 healthy preschool children, aged 18-60 months, evenly divided into two groups: caries-free (group I) and caries active (group II). Clinical features of ECC were meticulously inspected in each child, and the parents or caregivers completed the Child Behavior Checklist (CBCL), which comprises 100 questions related to a child’s daily behavior. The total scores, the narrow-band subscales, and raw scores were obtained. Accordingly, for each raw score, t-scores were obtained using the graphic display. These scores were then subjected to various statistical analyses including the Kolmogorov-Smirnov test, Mann-Whitney U test, and Spearman’s rank correlation method.

Results

Results of the present study revealed that there were no significant differences in behavior based on demographic factors such as gender and age. However notable differences were observed in several aspects of behavior between the two groups.

Conclusion

Caries-active children exhibited significantly higher levels of behavioral problems compared to their caries-free counterparts.

## Introduction

Early childhood caries (ECC) stands as one of the prevalent chronic infectious diseases of childhood and poses a significant public health challenge, presenting difficulties in its management and control [1]. It was known by various terms such as “nursing bottle syndrome,” “baby bottle tooth decay,” “bottle mouth caries,” “nursing caries,” “rampant caries,” “nursing bottle mouth,” “breast milk tooth decay” and “facio-lingual pattern of decay” in the past [2]. The American Academy of Pediatric Dentistry defines ECC as the presence of one or more decayed (noncavitated or cavitated) or filled tooth surfaces and/or missing teeth (because of caries), in a child under the age of six years old. For children aged three to five years old, one or more decayed, missing, or filled teeth, or a decayed, missing, or filled score of >4 (three years old), >5 (four years old), or >6 (five years old) teeth constitute severe ECC (sECC) [3].

The highest prevalence of ECC has been documented in the Far East Asian region, ranging from 36% to 85% among three-year-old children. In India, studies have reported a prevalence rate of 44% among children aged eight to 48 months [4,5]. Furthermore, rural areas of South India have shown a prevalence rate of 40.6% among children aged 0 to three years, with 50.3% exhibiting noncavitated surfaces, and 49.7% having cavitated surfaces [6]. The risk factors correlated with ECC encompass a spectrum of influences including dietary patterns, sociodemographic variables, oral hygiene practices, breastfeeding and bottle-feeding practices, microbial flora, and tooth structure as well as additional factors such as parental education levels and perinatal conditions. Parental knowledge greatly influences dietary habits and healthy behaviors. Socioeconomic constraints, particularly lower income levels, can hinder access to dental care, leading to the neglect of proper oral hygiene practices. Insufficient awareness about dental caries and inadequate maintenance of oral hygiene contribute to a higher prevalence of caries as effective prevention measures cannot be implemented [7]. Additionally, socioeconomic status, employment status of the mother, oral hygiene practices, and frequent medication use are identified as other risk factors contributing to this condition [8,9].

ECC has been demonstrated to affect the child’s overall well-being and significantly impact their quality of life [10]. It also leads to pain, speech problems, feeding difficulties, sleep problems, loss of school days, and psychological behavior changes in the child [11,12]. Previous literature has documented a connection between a child’s temperament and ECC [13]. Another study has reported that attention-deficit hyperactivity disorder (ADHD) has been related to dental caries [14]. Previous research has identified a correlation between dental caries and dental fear in children [15].

Child Behavior Checklist (CBCL) is a widely recognized and validated instrument utilized for the evaluation of behavioral and emotional issues in children and adolescents [16]. In dental research, CBCL has been employed to investigate the influence of emotional and behavioral problems on the selection of dental treatment and to explore the prevalence of dental anxiety among children from low-income backgrounds [17,18]. For instance, a study conducted by Williamson et al. [11] in Ohio utilized CBCL to explore the connection between ECC and children’s behavior.

However, as of our knowledge and to date, no studies have investigated the relationship between ECC and child behavior in the context of India. Therefore, the present study aims to bridge this gap by examining the association between ECC and behavioral changes in preschool children using a standardized assessment tool.

## Materials and methods

The present comparative cross-sectional study was approved by the Institutional Review Board (IRB) of the Sri Dharmasthala Manjunatheshwara (SDM) College of Dental Sciences and Hospital, Hubli, Dharwad, Karnataka, India. The study protocol was reviewed and approved by the Institutional Review Board (IRB) of the SDM College of Dental Sciences and Hospital, Hubli, Dharwad, Karnataka, India, recognized by the Dental Council of India, affiliated to Rajiv Gandhi University of Health Sciences, Bangalore, approval number IRB. No. 2014/P/PEDO/14. (It is now a deemed university.) We obtained informed consent from the parents or guardians of all the participants. About one hundred and twenty participants who visited the college department were selected using the convenience sampling technique and divided evenly into two groups based on clinical examination for both groups: group I (caries active (CA), 60 preschool children) and group II (caries-free (CF) preschool children, 60 preschool children).

The sample size was determined using the formula, n = Z2 × p × (1−p) / d2, where n = required sample size per group, Z = Z-score corresponding to the desired confidence level (e.g., 1.96 for a 95% confidence level), p = estimated proportion or prevalence of the characteristic or outcome of interest in the population, and d = desired margin of error or precision.

Preschool children aged 18-60 months of age, healthy (ASA I), and accompanied by a parent or caregiver who was able to complete the 100 questions of CBCL [16] were included in the study. Those children with a history of systemic disease or any physical disability and the parent/caregiver who were unable to consent to the study were excluded.

After the completion of the CBCL, the ratings were scored on the scales by the experienced specialist in the department, which included seven syndrome scales: (Emotionally Reactive (I), Anxious/Depressed (II), Somatic Complaints (III), Withdrawn (IV), Sleep Problems (V), Attention Problems (VI) and Aggressive Behavior (VII)) and five DSM scales (Depressive Problems (1), Anxiety Problems (2), Autism Spectrum Problems (3), Attention Deficit/Hyperactivity Problems (4), and Oppositional Defiant Problems (5)). The scores are 0 (not true), 1 (somewhat or sometimes true), or 2 (very true or often true) on the CBCL form filled by the parent were then transferred to the hand-scoring profile. Sixteen scores were reported as raw, and t-scores and normative values were established (normal behavior, borderline, and clinical problems). Higher t-scores showed more behavioral problems, with thresholds for borderline clinical and definitive clinical behaviors established [11].

The collected data were compiled and analyzed using Statistical Product and Service Solutions (SPSS, version 20.0; IBM SPSS Statistics for Windows, Armonk, NY) software. The following statistical tests were employed in our analysis: Kolmogorov-Smirnov test, Mann-Whitney U test, and Spearman’s rank correlation method. The level of significance was set at p < 0.05.

## Results

The present study included 120 participants evenly divided into two groups of 60 children each. There were 64 male and 56 female participants in this study. Most of the participants were aged between 49 and 60 months. Significant statistical difference was not observed between the groups about behavior based on demographics such as gender and age (Tables 1-2).

**Table 1:** Distribution of male and female children in the two study groups CA: caries active; CF: caries-free; %: percentage

Gender	CF group	%	CA group	%	Total	%
Male	36	60.00	28	46.67	64	53.33
Female	24	40.00	32	53.33	56	46.67
Total	60	100.00	60	100.00	120	100.00

**Table 2:** Distribution of children in the two study groups by age groups CA: caries active; CF: caries-free; %: percentage; SD: standard deviation

Age groups	CF group	%	CA group	%	Total	%
<=36 months	16	26.67	13	21.67	29	24.17
37–48 months	22	36.67	22	36.67	44	36.67
49–60 months	22	36.67	25	41.67	47	39.17
Total	60	100.00	60	100.00	120	100.00
Chi-square= 0.5032; P = 0.7783						
Mean age	46.17		46.85		46.51	
SD age	12.25		9.35		10.86	

It was observed that the mean and mean rank scores were higher in the caries-active (CA) group compared to CF and were statistically significant (p< 0.005) for all the domains (Table 3).

**Table 3:** Comparison of the two study groups (CF and CA) with respect to different variables by the Mann–Whitney U test *p<0.01; CA: caries active; CF: caries-free; SD: standard deviation

Variables	CF group			CA group			U-value	Z-value	P-value
	Mean	SD	Mean rank	Mean	SD	Mean rank			
Emotionally reactive	51.70	3.15	41.47	57.93	7.07	79.53	658.00	-5.9939	0.0001*
Anxious/depressed	51.27	2.19	39.08	59.02	8.73	81.92	515.00	-6.7445	0.0001*
Somatic problems	52.72	3.82	36.39	63.00	6.29	84.61	353.50	-7.5922	0.0001*
Withdrawn	52.03	4.48	41.52	59.80	9.42	79.48	661.00	-5.9782	0.0001*
Sleep problems	53.75	4.29	36.70	66.15	9.22	84.30	372.00	-7.4951	0.0001*
Attention problems	51.60	2.24	39.00	58.15	6.24	82.00	510.00	-6.7707	0.0001*
Aggressive behavior	50.12	0.42	36.99	56.65	6.33	84.01	389.50	-7.4032	0.0001*
Internalizing problems	44.70	6.43	34.30	60.53	8.51	86.70	228.00	-8.2509	0.0001*
Externalizing problems	41.68	4.50	33.26	56.22	7.58	87.74	165.50	-8.5789	0.0001*
Total problems	33.92	3.68	35.23	43.13	7.65	85.78	283.50	-7.9596	0.0001*
Depressive problems	51.37	3.26	35.30	62.07	8.76	85.70	288.00	-7.9359	0.0001*
Anxiety problems	52.95	3.56	41.03	62.27	9.49	79.98	631.50	-6.1330	0.0001*
Autism spectrum problems	51.88	3.22	39.82	61.38	8.84	81.18	559.00	-6.5136	0.0001*
Attention deficit/hyperactivity problems	51.20	1.92	37.57	59.47	6.76	83.43	424.00	-7.2221	0.0001*
Appositional defiant problems	50.27	0.80	37.32	57.98	7.72	83.68	409.00	-7.3009	0.0001*

Some domains within CBCL of group I showed a negative correlation (Table 4).

**Table 4:** Correlations among all the variables in the CF group by the Spearman’s rank correlation method *p<0.05, CF: caries-free

Variable	Summary	X1	X2	X3	X4	X5	X6	X7	X8	X9	X10	X11	X12	X13	X14	X15
X1	Spearman’s R	-														
	P value	-														
X2	Spearman’s R	0.1360	-													
	P value	0.3010	-													
X3	Spearman’s R	-0.0190	0.1150	-												
	P value	0.8850	0.3830	-												
X4	Spearman’s R	0.0120	0.2210	0.0360	-											
	P value	0.9290	0.0900	0.7840	-											
X5	Spearman’s R	0.2180	0.0300	0.0330	0.0620	-										
	P value	0.0940	0.8210	0.8020	0.6370	-										
X6	Spearman’s R	-0.0020	0.1550	0.0950	0.0790	0.2150	-									
	P value	0.9890	0.2380	0.4690	0.5500	0.0980	-									
X7	Spearman’s R	0.2900	0.1020	0.1350	-0.0160	-0.0510	0.0630	-								
	P value	0.0250*	0.4370	0.3050	0.9030	0.6970	0.6330	-								
X8	Spearman’s R	0.4300	0.6490	0.5180	0.4420	0.1690	0.1990	0.2040	-							
	P value	0.0010*	0.0001*	0.0001*	0.0001*	0.1980	0.1280	0.1170	-							
X9	Spearman’s R	0.1100	0.1460	0.2630	0.0880	0.0730	0.6330	0.4530	0.2710	-						
	P value	0.4050	0.2670	0.0420*	0.5060	0.5790	0.0001*	0.0001*	0.0360*	-						
X10	Spearman’s R	0.3570	0.1120	0.3240	0.0320	0.6450	0.0890	0.0880	0.4570	0.1650	-					
	P value	0.0050*	0.3950	0.0120*	0.8060	0.0001*	0.5000	0.5020	0.0001*	0.2070	-					
X11	Spearman’s R	0.1250	0.0020	0.0450	0.1150	0.3100	0.1390	-0.1060	0.0970	-0.0610	0.1820	-				
	P value	0.3420	0.9890	0.7300	0.3820	0.0160*	0.2890	0.4200	0.4610	0.6440	0.1640	-				
X12	Spearman’s R	0.3620	0.2630	0.3160	0.0830	0.3430	-0.0820	0.1360	0.4850	-0.0490	0.6020	0.0030	-			
	P value	0.0040*	0.0420*	0.0140*	0.5280	0.0070*	0.5350	0.3020	0.0001*	0.7100	0.0001*	0.9810	-			
X13	Spearman’s R	0.3580	0.0480	0.1740	0.4160	0.4420	0.0980	0.1870	0.4390	0.2110	0.4680	0.0210	0.2350	-		
	P value	0.0050*	0.7170	0.1830	0.0010*	0.0001*	0.4570	0.1520	0.0001*	0.1060	0.0001*	0.8750	0.0710	-		
X14	Spearman’s R	0.2650	0.1970	0.2120	-0.1180	0.1220	0.4320	0.4410	0.2410	0.7240	0.1970	-0.0470	0.0450	0.0720	-	
	P value	0.0400*	0.1310	0.1040	0.3680	0.3510	0.0010*	0.0001*	0.0640	0.0001*	0.1320	0.7220	0.7330	0.5860	-	
X15	Spearman’s R	0.2990	0.1810	0.3810	0.2600	0.0630	0.1770	0.6090	0.4300	0.5480	0.2710	-0.0890	0.3190	0.3780	0.3790	-
	P value	0.0200*	0.1670	0.0030*	0.0450*	0.6350	0.1770	0.0001*	0.0010*	0.0001*	0.0370*	0.5010	0.0130*	0.0030*	0.003*	-

While the majority showed a positive correlation. However, all domains in group II showed a positive correlation (Table 5).

**Table 5:** Correlations among all the variables in the CA group by Spearman’s rank correlation method *p<0.05, CA: caries active

Variables	Summary	X1	X2	X3	X4	X5	X6	X7	X8	X9	X10	X11	X12	X13	X14	X15
X1	Spearman’s R	-														
	P value	-														
X2	Spearman’s R	0.6880	-													
	P value	0.0001*	-													
X3	Spearman’s R	0.5280	0.3370	-												
	P value	0.0001*	0.0080*	-												
X4	Spearman’s R	0.6680	0.7120	0.2550	-											
	P value	0.0001*	0.0001*	0.0490	-											
X5	Spearman’s R	0.3420	0.2490	0.3700	0.2190	-										
	P value	0.0070*	0.0550	0.0040*	0.0930	-										
X6	Spearman’s R	0.5330	0.5520	0.2530	0.5080	0.3570	-									
	P value	0.0001*	0.0001*	0.0510	0.0001*	0.0050*	-									
X7	Spearman’s R	0.6460	0.5960	0.3820	0.5830	0.4100	0.6410	-								
	P value	0.0001*	0.0001*	0.0030*	0.0001*	0.0010*	0.0001*	-								
X8	Spearman’s R	0.8840	0.8590	0.6320	0.7930	0.3590	0.5760	0.6980	-							
	P value	0.0001*	0.0001*	0.0001*	0.0001*	0.0050*	0.0001*	0.0001*	-							
X9	Spearman’s R	0.6750	0.6220	0.3980	0.6270	0.4250	0.7690	0.9730	0.7270	-						
	P value	0.0001*	0.0001*	0.0020*	0.0001*	0.0010*	0.0001*	0.0001*	0.0001*	-						
X10	Spearman’s R	0.7130	0.6740	0.4600	0.6590	0.5650	0.5460	0.6310	0.7730	0.6770	-					
	P value	0.0001*	0.0001*	0.0001*	0.0001*	0.0001*	0.0001*	0.0001*	0.0001*	0.0001*	-					
X11	Spearman’s R	0.6300	0.6810	0.3850	0.6470	0.4830	0.6300	0.6250	0.7560	0.6790	0.6850	-				
	P value	0.0001*	0.0001*	0.0020*	0.0001*	0.0001*	0.0001*	0.0001*	0.0001*	0.0001*	0.0001*	-				
X12	Spearman’s R	0.6400	0.7000	0.5570	0.5700	0.4600	0.5190	0.5330	0.7530	0.5720	0.6620	0.5990	-			
	P value	0.0001*	0.0001*	0.0001*	0.0001*	0.0001*	0.0001*	0.0001*	0.0001*	0.0001*	0.0001*	0.0001*	-			
X13	Spearman’s R	0.7120	0.5860	0.5520	0.7900	0.2400	0.4110	0.5290	0.7830	0.5590	0.6730	0.5180	0.5700	-		
	P value	0.0001*	0.0001*	0.0001*	0.0001*	0.0650	0.0010*	0.0001*	0.0001*	0.0001*	0.0001*	0.0001*	0.0001*	-		
X14	Spearman’s R	0.3820	0.4190	0.2150	0.4040	0.3700	0.8030	0.6100	0.4590	0.7060	0.5160	0.5940	0.3770	0.3330	-	
	P value	0.0030*	0.0010*	0.0990	0.0010*	0.0040*	0.0001*	0.0001*	0.0001*	0.0001*	0.0001*	0.0001*	0.0030*	0.0090*	-	
X15	Spearman’s R	0.5850	0.4720	0.5660	0.5120	0.5360	0.6050	0.7840	0.6520	0.8030	0.5950	0.6490	0.5800	0.4750	0.5880	-
	P value	0.0001*	0.0001*	0.0001*	0.0001*	0.0001*	0.0001*	0.0001*	0.0001*	0.0001*	0.0001*	0.0001*	0.0001*	0.0001*	0.0001*	-

## Discussion

The detrimental effects of dental caries on dental and general health are well known. In recent years, there has been a growing focus on addressing the emotional, social, and behavioral challenges faced by the affected children and their families [19]. Hence, a validated instrument such as CBCL was used in the present study to assess child behavior. The CBCL is a key component of the Achenbach System of Empirically Based Assessment (ASEBA), designed to identify and assess the behavioral and emotional issues in children and adolescents. It provides the benefit of conducting a comprehensive assessment of behavioral problems, even in children with medical and various other conditions [20].

Similar to the results of the present study, Williamson et al.’s [11] study did not reveal significant differences between the two groups in terms of demographic factors such as gender and age. Various behavioral areas such as emotional reactive, somatic problems, withdrawal, sleep problems, attention problems, anxiety/depression, aggressive behavior, internalizing problems, externalizing problems, depressive problems, anxiety problems, total problems, autism spectrum problems, attention-deficit/hyperactivity problems, and oppositional defiant problems in the present study showed statistically significant differences between both groups, with more behavioral problems in group II, which is similar to findings observed in earlier studies. An association between the temperament of the child and ECC was reported in some studies, while other studies reported behavior disruption in children with dental pain [13,21,22]. Easton et al. [23] conducted a study that reported differences in child behavior before dental visits were different for children with significant ECC compared to their caries-free counterparts, which is under this study.

Findings based on child temperament suggest that ECC was prominent in the shy and withdrawn child compared to the caries-free group, which is under the present study [18,24]. Similarly, another study [25] found that children with dental caries exhibited lower levels of sociability, reduced energy, increased distractibility, moderate emotional expression, and a decreased sense of rhythmicity. The authors also showed that disturbed sleep and sleep deprivation because of dental caries may contribute to such behavior, which aligns with the results of this study. Recent research conducted in Japan found an association between the poor sleeping quality of both children and parents and the prevalence of dental caries among preschool-aged children [26]. A study conducted in Columbus, Ohio, which used CBCL reported statistically higher scores for sleep problems in participants with ECC [11]. Similarly, the mothers of children in the caries group reported higher use of bottle-feeding as a method to address sleep issues, which was more common in children with dental caries than in the comparison group. It was demonstrated that the presence of sleep problems was related to the use of sleep-associated feeding as a means of sleep management [12]. Marino et al. [27] also noted that children with nursing caries and strong tempers were more likely to have sleep difficulties than those without dental disease.

In a previous study [11], significantly higher scores were observed for anxiety, depression, externalizing problems, aggressive behavior, and attention deficit-hyperactivity problems in the CBCL, aligning with the findings of the present study. It has been reported that factors such as marital violence, dysfunctional parenting styles, and maternal stress may serve as contributing factors to these behavioral patterns. Additionally, the children of mothers experiencing high levels of stress have been observed to be at an increased risk for poor parenting, neglect, and abuse [28]. In a study utilizing the International Caries Detection and Assessment System (ICDAS), it was revealed that children affected by ADHD had an elevated risk of developing dental caries in both their primary and permanent dentition [29]. A recent meta-analysis further supported these findings, indicating that children with ADHD were more susceptible to dental caries development compared to their healthy peers [30]. Similar outcomes were observed in the current study. However, it is crucial to acknowledge that dietary habits and insufficient oral hygiene also contribute to the increased risk of decay in ADHD children [29].

The present study has several limitations. Although a strong association between ECC and behavioral problems was found in this study, it is unclear whether the behavioral changes started first or ECC. Second, the findings of the present study were obtained from the participants who visited a single institution. Hence, the results cannot be generalized. Third, the medical reports of the participants were not verified, and parental verbal affirmation was considered. Further research is required to know the extent of the influence of the ECC on the well-being of children.

## Conclusions

The results of the present study showed significant disparities in the behavior of children with and without dental caries. When considering demographic factors such as gender and age, there were no significant statistical variances in the behavior of preschool children about the presence or absence of dental caries.

Significant variation was observed between the two groups: group I (CF) and group II (CA) in the present investigation, which encompassed a wide array of behavioral dimensions including: emotionally reactive, somatic problems, anxious/depressed, withdrawn, sleep problems, attention problems, aggressive behavior, internalizing problems, externalizing problems, total problems, depressive problems, anxiety problems, autism spectrum problems, ADHD, and oppositional defiant problems. Crucially these disparities were statistically significant. These pieces of evidence suggest a strong relationship between ECC and behavioral changes in children, which highlights the intricate interplay between oral health and the behavioral well-being of preschool-aged children.
